# Single Cell Analysis Identifies the miRNA Expression Profile of a Subpopulation of Muscle Precursor Cells Unique to Humans With Type 2 Diabetes

**DOI:** 10.3389/fphys.2018.00883

**Published:** 2018-07-11

**Authors:** Tora I. Henriksen, Sarah E. Heywood, Ninna S. Hansen, Bente K. Pedersen, Camilla C. Scheele, Søren Nielsen

**Affiliations:** ^1^Centre for Inflammation and Metabolism and the Centre for Physical Activity Research, Rigshospitalet, University of Copenhagen, Copenhagen, Denmark; ^2^The Novo Nordisk Foundation Center for Basic Metabolic Research, Faculty of Health and Medical Sciences, University of Copenhagen, Copenhagen, Denmark

**Keywords:** diabetes, human, microRNA, muscle, satellite cell, single-cell analysis, muscle stem cells

## Abstract

MicroRNAs (miRNAs) take part in regulating central cellular processes such as differentiation and metabolism. We have previously shown that muscle progenitor cells derived from individuals with type 2 diabetes (T2DM) have a dysregulated miRNA profile. We hypothesized that the T2DM muscle progenitor population is heterogeneous in its miRNA expression and differs from the progenitor population of healthy controls. MiRNA expression profiles of CD56+ muscle progenitor cells from people with T2DM and from healthy controls were therefore investigated at a single cell level. Single-cell analysis revealed three subpopulations expressing distinct miRNA profiles: two subpopulations including both T2DM and healthy control muscle precursors presented miRNA expression profiles mostly overlapping between groups. A distinct third subpopulation consisted solely of cells from donors with T2DM and showed enriched expression of miRNAs previously shown to be associated with type 2 diabetes. Among the enriched miRNAs was miR-29, a regulator of *GLUT4* mRNA expression. Interestingly, this subpopulation also revealed several miRNAs with predicted targets in the PI3K/Akt pathway, not previously described in relation to T2DM muscle dysfunction. We concluded that a subpopulation of T2DM muscle precursor cells is severely dysregulated in terms of their miRNA expression, and accumulation of this population might thus contribute to the dysfunctional muscular phenotype in type 2 diabetes.

## Introduction

Skeletal muscle is a major target for insulin-stimulated glucose uptake, and dysregulated muscle metabolism is a hallmark of type 2 diabetes (T2DM) (Czech, 2017). MicroRNAs (miRNAs) are short, non-coding RNAs that post-transcriptionally modulate gene expression (Lee et al., 1993; Lim et al., 2005) and have been shown to regulate metabolism and insulin sensitivity (Frost and Olson, 2011; Trajkovski et al., 2011; Kornfeld et al., 2013). Accordingly, multiple disease pathologies are associated with aberrant miRNA expressions, including type 2 diabetes (He et al., 2017).

Skeletal muscle expresses augmented levels of a number of miRNAs (Sempere et al., 2004) which regulate critical muscle cell processes, such as differentiation and mitochondrial biogenesis (Chen et al., 2006; Yamamoto et al., 2012). Considering the central role of muscle dysfunction in T2DM, it is not surprising that skeletal muscle of patients with T2DM presents a dysregulated miRNA expression profile (Gallagher et al., 2010; Bork-Jensen et al., 2015). In line with this, we recently showed that isolated muscle stem cells from T2DM donors have a discordant miRNA expression profile compared with cells from healthy controls, resulting in impaired myogenesis (Henriksen et al., 2017). However, out of approximately 1000 analyzed miRNAs, we only found 5 miRNAs to be differentially expressed in the T2DM cells, whereas the expression of a number of miRNAs were just below the threshold for statistical significance when corrected for multiple testing (Henriksen et al., 2017). In addition, several of the borderline significant miRNAs could be localized to genomic clusters also hosting the five significantly altered transcripts. A heterogeneous cell population could provide one explanation for the observed blunted disease phenotype, since measuring an average based on a whole cell population can mask relevant expression patterns in underlying subpopulations of cells (Altschuler and Wu, 2010). Moreover, subpopulation trends may disappear or appear reversed when combined into an ensemble measurement, as explained by Simpson’s Paradox (Simpson, 1951).

Skeletal muscle progenitor cells (satellite cells) represent just one player in a heterogeneous stem cell niche that also includes fibroblasts (Agley et al., 2015). Besides the interaction between the two cells types (Mackey et al., 2017), the stem cell microenvironments are also affected by other factors such as hormones, metabolites and biomechanical forces (Gattazzo et al., 2014). All of these are factors that are altered in a type 2 diabetic state and can thereby modulate the stem cell phenotype (Park et al., 2006; Lewitt et al., 2014; Molnos et al., 2017). It is therefore likely that muscle progenitor cells represent a heterogeneous cell group with distinct cellular subpopulations, and likewise may encompass discreet myoblast subpopulations with similarly diverse miRNA expression profiles.

Our previous finding that muscle progenitor cells from T2DM donors only express a low number of significantly differently expressed miRNAs compared with healthy donors might thus be due to averaging miRNA expressions in a heterogeneous cell population. With the present study, we therefore address this issue at a single-cell level by sorting individual CD56+ muscle progenitor cells from people with T2DM and healthy control subjects, and analyzing miRNAs previously reported to be borderline significantly expressed between the two groups (Henriksen et al., 2017). We hypothesize that human muscle progenitor cells have cellular subpopulations with distinct miRNA expression profiles, and that muscle progenitor cells from T2DM donors have distinct miRNA expression profiles compared with healthy donors.

## Research Design and Methods

### Human Subjects

Cell cultures were obtained from male donors (**Table 1**) included in a previously described study (Pedersen et al., 2012). A total of 3 of the 5 diabetic donors were taking a combination of Simvastatin and either Metformin or Acetylsalicylic acid; one donor was taking Centyl (a diuretic) and an ACE-inhibitor in addition to Simvastatin and Metformin. To minimize the effect of different treatments participants with type 2 diabetes were not allowed taking any antidiabetic medication for one week before meeting at the hospital. None of the diabetic donors received insulin as medication. All participants gave written informed consent before inclusion. The study was performed according to the Declaration of Helsinki and approved by The Regional Committee on Biomedical Research Ethics in Denmark (KF 01-141/04).

**Table 1:** Clinical characteristics of muscle precursor cell donors.

	Healthy (*n* = 5)	T2DM (*n* = 5)
Age (years)	55.0 (50–59)	58 (52–66)
BMI (kg/m^2^)	23.8 ± 1.3	27.4 ± 0.9^∗∗^
Fasting glucose (mmol/L)	5.1 ± 0.6	9.4 ± 3.4^∗^
OGTT 2-h glucose (mmol/L)	5.5 ± 0.8	17.9 ± 6.3^∗∗^
Fasting insulin (pmol/L)	42.2 ± 11.6	61.4 ± 17.1
OGTT 2-h insulin (pmol/L)	305.8 ± 197.6	223 ± 161.9
HOMA-IR	1.6 ± 0.5	4.3 ± 1.9^∗^
VO_2_ max (L/min)	2.9 ± 0.7	2.4 ± 0.7
VO_2_ max (mL/min/kg)	35.9 ± 9.9	26.8 ± 6.4

*Data are means ± SE. BMI = body mass index; OGTT = oral glucose tolerance test. Glucose values are mmol/L, and insulin values are pmol/L. Differences between groups were compared using Student’s unpaired t-test. ^∗^P < 0.05, ^∗∗^P < 0.001, ^∗∗∗∗^P < 0.0001.*

### Materials

F10 nutrient mixture (HAM), Dulbecco’s modified Eagle’s medium (DMEM), fetal bovine serum (FBS), horse serum (HS), penicillin/streptomycin (P/S), and Fungizone antimycotic (FZ) were obtained from Invitrogen (Taastrup, Denmark). CD56-conjugated microbeads were from Miltenyi Biotec (Lund, Sweden). C1^TM^ System and C1 integrated fluidic circuits (IFCs) were from Fluidigm (San Francisco, CA, United States). LIVE/DEAD cell staining solution, ActinRed and NucBlue ReadyProbes antibodies, and Megaplex PreAmp primers were from Thermo Scientific (Taastrup, Denmark).

### Microarray

As previously described (Henriksen et al., 2017), a miRCURY LNA microRNA Array (sixth gen – hsa, mmu, and rno) (Exiqon, Denmark) was utilized for global miRNA detection between human muscle stem cells derived from T2DM subjects and healthy controls during differentiation (GSE86069).

### Human Muscle Satellite Cell Isolation and Culture

Satellite cells were isolated from *vastus lateralis* muscle biopsies as previously described (Green et al., 2011). After removal of fat and connective tissue, the muscle biopsy was minced into small pieces and digested in buffer containing 0.05% trypsin-EDTA, 1 mg/ml collagenase IV and 10 mg/ml BSA for 5 min at 37°C. Subsequently, digestion solution containing liberated muscle precursor cells was transferred to cold FBS to stop trypsin activity. The solution was centrifuged at 800 g for 7 min. The supernatant was removed and washed with F10/HAM. To minimize fibroblast contamination, the cell suspension was pre-plated in a culture plate for 3 h in growth medium containing 20% FBS, 1% PS, and 1% FZ in F10/HAM. The unattached cells were seeded onto Matrigel coated culture flasks (0.01% Matrigel in F10/HAM, 30 min at 37°C) and cultured for 4 days in growth medium in a humidified incubator with 5% O_2_ and 5% CO_2_ at 37°C. After 4 days of incubation, cell culture medium was changed and then every second day thereafter. All experiments were performed on myoblasts at passage 1–2.

### Immunomagnetic Sorting of CD56+ Cells

Cells were sorted for the cell surface marker CD56 using immunomagnetic column sorting (MACS) to achieve pure populations of muscle precursor cells, as described by Agley et al. (2015). Cells grown to ∼50% confluency in a 10 cm culture dish were incubated with Human CD56 primary antibody conjugated magnetic microbeads (Miltenyi Biotec) at 4°C for 30 min. CD56+ myoblasts were filtered from the bulk population using a magnetic cell separator (Miltenyi Biotec) according to the manufacturer’s instructions (Miltenyi Biotec).

### Single Cell miRNA Amplification

Single cell capture, specific reverse transcription of miRNAs, and cDNA pre-amplification were performed using the Fluidigm^®^ C1^TM^ System. The cells were loaded in the C1^TM^ Single-Cell Preamp IFC, for cell size 10–17 μm (Fluidigm) according to the manufacturer’s protocol (PN 100-6667). Pre-amplification was performed using Megaplex PreAmp Pool A primers (Thermo Scientific) and Single Cell PreAmp Mix (Ambion). Cells were stained with a LIVE/DEAD fluorescent assay (Thermo Scientific) to identify presence of living cells. All cell capture sites were manually inspected on an EVOS FL fluorescent microscope (Thermo Scientific); capture sites containing debris, multiple or dead cells, or no cells were excluded from further analysis (**Figure 2C** and **Table 2**).

**Table 2:** IFC cell capture rates.

	Healthy					T2DM					Total	
Donors	32a	40a	92a	21a	99a	129a	113a	7a	27a	48a	Healthy	T2DM
Captured	83	86	84	25	43	87	46	79	14	88	321	314
Live	56	62	70	19	37	74	36	64	14	72	244	260
No Capture	13	10	12	71	53	9	50	17	82	8	159	166
Dead	4	2	1	1	0	1	0	3	0	2	8	6
Debris	15	19	10	3	1	4	5	4	0	10	48	23
Multiple	8	3	3	2	5	8	5	8	0	4	21	25
Total	96	96	96	96	96	96	96	96	96	96	480	480

*Individual quality control scoring of capture sites in single cell 96 capture site IFCs.*

### RT-PCR

Targeted miRNA expression analysis was performed using a gene expression Dynamic Array^TM^ 48.48 IFC (PN 68000130) on an MX IFC controller and the BioMark HD System for real-time PCR (RT-PCR; Fluidigm). TaqMan miRNA primer assays and TaqMan Universal PCR Master Mix, no AmpErase UNG was used for the qPCR (Thermo Scientific). A total of 34 miRNAs were selected for analysis based on their relative expression between healthy and T2DM groups in a previous study (Henriksen et al., 2017); the included miRNAs are listed in **Table 3**.

**Table 3:** Differentially expressed miRNA.

	*t*-test (*P*-values)				log2 fold change			
miRNA	T2DM vs. T2DM group 2	Healthy vs. T2DM group 2	Healthy vs. T2DM	Healthy vs. Mix	T2DM vs. T2DM group 2	Healthy vs. T2DM group 2	Healthy vs. T2DM	Healthy vs. Mix
miR-24	1,7E-59	2,7E-74	8,8E-06	1,8E-15	-5,5	-6,1	-0,7	2,7
miR-191	7,7E-38	2,7E-77	9,5E-01	7,4E-27	-7,0	-7,7	-0,8	3,6
miR-29c	7,2E-36	6,0E-38	7,5E-01	5,6E-08	-6,9	-6,9	0,0	3,6
miR-29b	6,0E-30	9,7E-34	5,5E-01	5,0E-01	-11,5	-12,8	-1,2	9,4
miR-210	2,1E-23	1,5E-25	6,0E-02	2,4E-04	-9,4	-8,4	0,9	5,6
miR-29a	2,1E-22	4,6E-56	1,7E-04	7,7E-22	-4,8	-5,2	-0,4	3,6
miR-17	6,7E-19	1,2E-37	5,7E-04	1,8E-32	-5,7	-5,4	0,3	6,1
miR-19b	3,8E-17	1,2E-24	7,8E-01	8,5E-14	-5,6	-5,8	-0,2	2,3
miR-27b	9,7E-17	2,8E-14	2,3E-01	8,7E-03	-6,3	-6,4	-0,1	15,1
miR-193b	2,2E-16	8,0E-19	2,9E-03	1,8E-05	-5,6	-5,9	-0,3	3,8
miR-199a-3p	1,8E-14	1,6E-13	1,6E-01	1,4E-08	-6,7	-7,0	-0,3	7,5
miR-138	8,3E-07	2,0E-07	4,8E-01	3,3E-03	-1,1	-1,4	-0,3	1,5
miR-30c	3,5E-05	2,4E-05	5,0E-01	1,5E-12	-4,9	-5,2	-0,3	6,1
miR-125b	3,6E-05	3,6E-05	8,6E-01	2,8E-13	-7,2	-7,3	-0,1	14,7
miR-365	1,7E-04	3,0E-04	6,7E-01	9,6E-05	-5,0	-5,6	-0,6	1,9
miR-214	4,1E-04	4,7E-04	2,7E-01	3,6E-06	-6,0	-6,1	-0,1	2,6
miR-20a	2,2E-03	3,5E-03	6,7E-01	3,1E-06	-5,3	-5,4	-0,2	7,7
miR-27a	1,4E-02	3,4E-02	9,0E-02	2,2E-07	-5,0	-4,6	0,4	2,7
miR-145	1,9E-02	2,2E-02	1,7E-03	3,4E-02	-8,5	-6,2	2,3	3,7
miR-106b	7,9E-02	5,6E-02	2,7E-01	1,7E-03	-3,7	-3,5	0,2	15,1
miR-195	1,0E-01	7,1E-02	6,2E-01	3,6E-04	-2,8	-3,1	-0,3	11,5
miR-30b	1,1E-01	2,5E-01	9,0E-01	8,1E-08	-4,8	-5,6	-0,8	2,8
miR-10a	1,2E-01	7,5E-02	7,0E-03	2,6E-01	-9,6	-7,6	2,0	11,7
miR-143	5,9E-01	3,8E-01	5,9E-02	1,3E-01	-4,3	-4,5	-0,3	12,3
miR-140-3p	6,2E-01	5,7E-01	3,0E-01	1,8E-01	-3,3	-3,4	-0,1	8,7
miR-708	9,6E-01	9,2E-01	8,3E-01	6,7E-04	-4,2	-4,1	0,1	2,9

*Differences between groups were compared using Student’s unpaired t-test with Bonferroni’s correction for multiple testing. Significant inter-group differences are highlighted in green.*

### FACS

Isolated muscle precursor cells were propagated in growth medium as described above until 70% confluence. Cells were detached using TrypLE^TM^ Express, and subsequently washed twice in FACS buffer [phosphate-buffered saline (PBS) containing 2% heat inactivated FBS]. Cells were stained with anti-human CD56-APC, CD31-PE, and CD45-BV421 (all from BD Bioscience) for 20 min and subsequently washed twice in FACS buffer. Data was acquired using a FACS Fortessa (BD Biosciences). For compensation, single stain was used with one drop of negative control beads and anti-mouse IgG beads (BD Biosciences). Data analysis was performed using FlowJo software version 10.

### Pathway Prediction and miRNA Target Gene Analysis

The miRNA target pathway predictions were performed using Diana miRPath software (Vlachos et al., 2012) based on TargetScan analysis. Context score was set to 0.4, *P*-value was set to 0.05, and conservative stats were applied. To visualize miRNA-gene interactions the CytoScape app CyTargetLinker was used (Shannon et al., 2003; Kutmon et al., 2013). Regulatory interaction networks comprising miRNA-target interactions were obtained from http://projects.bigcat.unimaas.nl/cytargetlinker/regins. Validated targets (miRTarBase) and predicted targets (MicroCosm and TargetScan) were integrated and overlap threshold was set to 1. The resulting interaction network was crosschecked for genes within the PI3K/Akt signaling pathway.

### Statistical Analyses

The RT-qPCR data was pre-processed on the Biomark software (Fluidigm). Threshold (0.1) and baseline were adjusted for comparison across the IFC qPCR plates. A quality control score at 0.6 (QC feature in Biomark software) was defined as a successful signal. All live cells expressing RNU48 were included in the analysis. miRNA qPCR assays with an efficiency higher than 10% were used as an inclusion criterion. All dead cells, multiple cells and debris were filtered and excluded from the data. Relative values were then calculated using the standard curve method. Expression levels are presented per cell and not normalized to a reference gene as when performing bulk qPCR analysis. To identify differentially expressed miRNAs between groups, an unpaired *t*-test was performed. *P*-values (0.05) were corrected for multiple comparisons using the Bonferroni’s correction approach. To visualize clustering of multivariate data, miRNA qPCR data was log-transformed and uploaded to the web-based bioinformatic tool ClustVis (Metsalu and Vilo, 2015). A principal component analysis (PCA) plot (PC1: 28.9% and PC2: 6.7%) was generated to identify unique clusters within the two groups. After identification of clusters, data was classified in four unique groups and a heat map (unsupervised clustering) was performed. The method for clustering rows and columns was ‘average’ and a Manhattan approach was used to visualize column distance.

## Results

**FIGURE 1:**
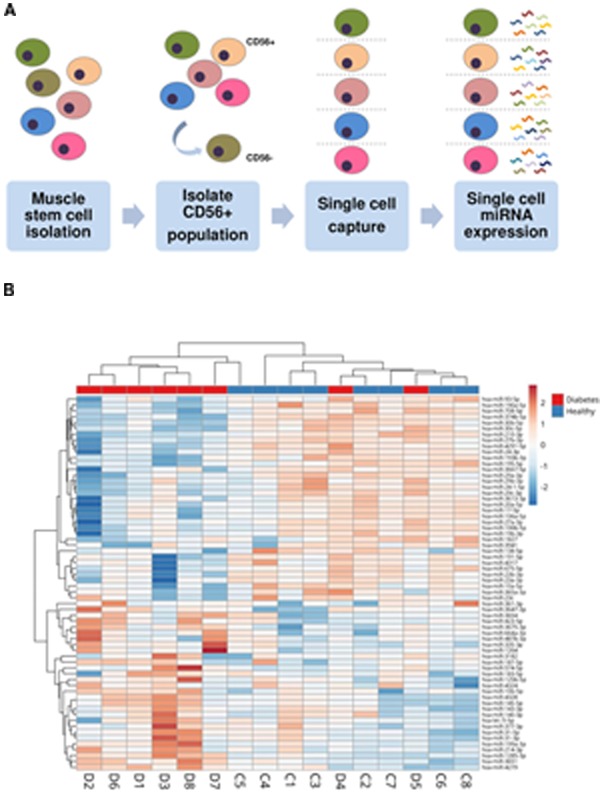
**(A)** Schematic overview of study workflow. Muscle precursor cells included in the present study were isolated from healthy (*n* = 5) or T2DM (*n* = 5) donors (donor characteristics are summarized in **Table 1**). Proliferating myoblasts expressing the myoblast marker CD56 were positively selected for further analysis. Individual cells were isolated through use of single-cell microfluidics and assessed for their respective miRNA expression profiles. **(B)** Heat map of bulk miRNA expression in healthy versus T2DM proliferating muscle precursors. This is a subset of data previously described (Henriksen et al., 2017).

A schematic overview of the study workflow is seen in **Figure 1A**. A global ensemble miRNA analysis previously performed in differentiating muscle precursor cells from healthy and T2DM donors showed that a subset of miRNAs tended to be differentially regulated in differentiating T2DM muscle precursors (Henriksen et al., 2017). Unsupervised clustering analysis of this data set revealed a tendency for group-based clustering, however, this varied considerably between cells from individual donors (**Figure 1B**), suggesting heterogeneity of miRNA expression within cells of each group. We therefore hypothesized that the miRNA expression of individual cells would vary between groups. To address this hypothesis, we selected 34 miRNAs identified as either significant or borderline significantly expressed between healthy and T2DM groups (Henriksen et al., 2017) for single-cell analysis in the present study. The miRNA included for single-cell analysis are listed in **Table 3** and the miRNA assay detection rates in the present study are shown in **Figure 3A**.

**FIGURE 2:**
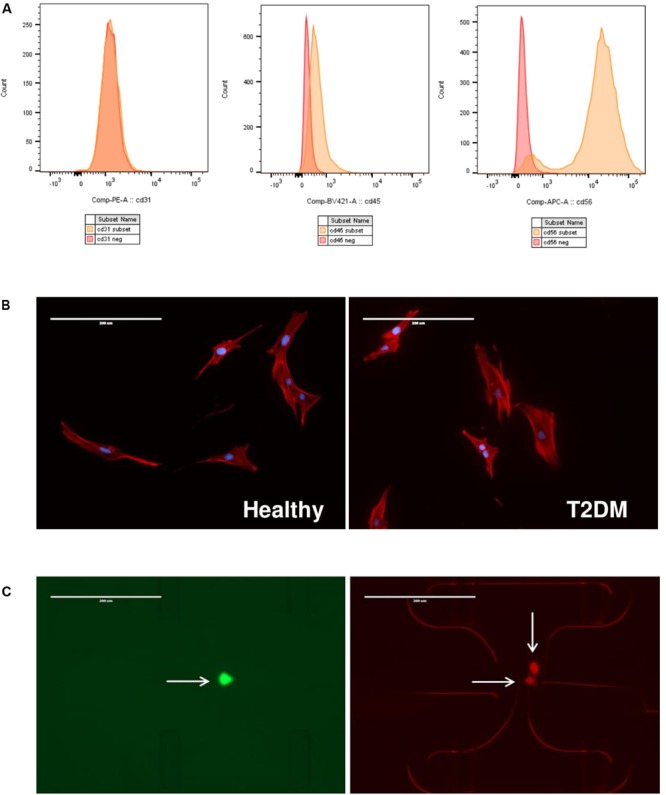
**(A)** Flow cytometry analysis of healthy and T2DM muscle precursor cells. Representative histograms for positive (orange) and negative (red) cell subsets: CD31 conjugated to PE; CD45 conjugated to BV421 and CD56 conjugated to APC. **(B)** Immunofluorescence images of cells from healthy (left panel) or T2DM donors (right panel) stained for actin (red) or nucleus (blue). Scale bar indicates 400 μm. Representative images are shown. **(C)** Immunofluorescence images of IFC capture sites containing single, live cell stained with Calcein-AM (left image; green) or multiple dead cells stained with ethidium-homodimer 1 (right image; red). Scale bar indicates 400 μm.

**FIGURE 3:**
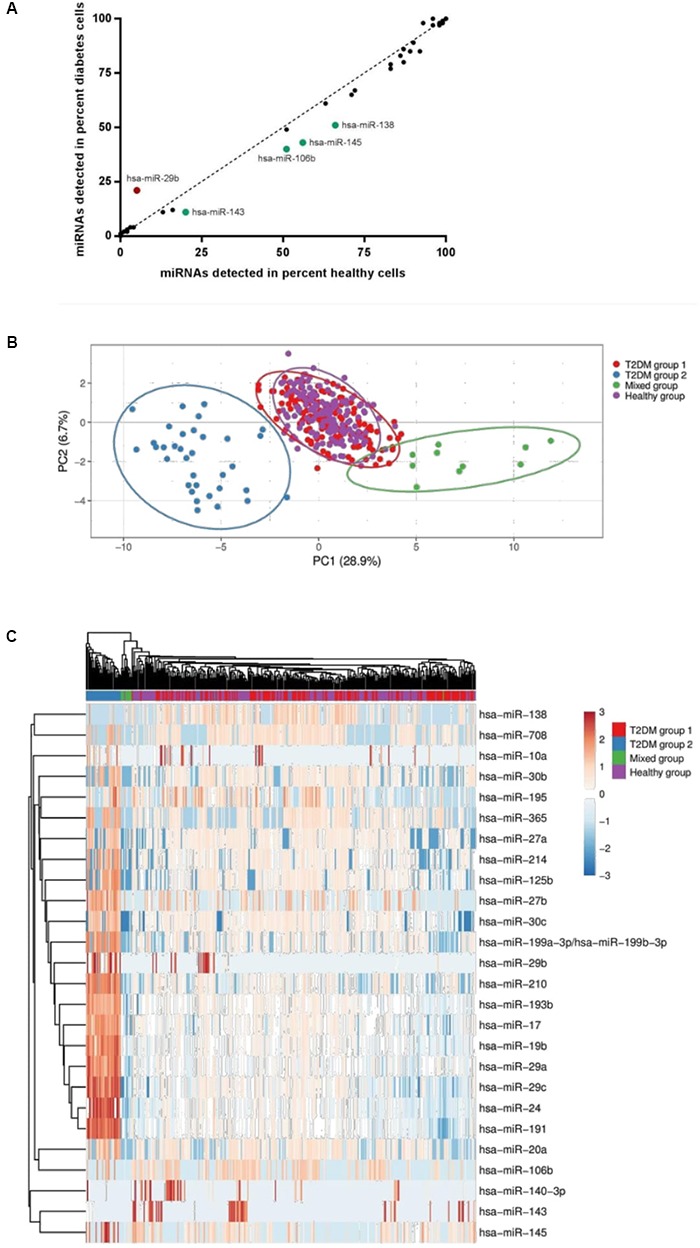
**(A)** miRNA detection rates in healthy versus T2DM muscle precursor cells. miRNA detected to a higher degree in T2DM cells are highlighted in red; miRNA detected to a higher degree in healthy cells are highlighted in green. **(B)** Principal component analysis of single-cell miRNA expression in the four defined groups (Healthy, Mixed, T2DM group 1 and T2DM group 2). **(C)** A heat map including the miRNA expression for all four groups defined by PCA, using an unsupervised clustering approach.

Primary myoblasts from healthy or type 2 diabetic donors were subjected to immunomagnetic column sorting to positively select for CD56 as a marker of myogenic cells; clinical characteristics of cell donors are shown in **Table 1**. We subsequently performed FACS analysis of a subset of cells to verify myogenic purity of cell cultures. The analyzed cells did not express endothelial marker CD31 or hematopoietic marker CD45, confirming the absence of endothelial and immune cells in the cell cultures (**Figure 2A**). FACS analysis showed that all analyzed cell cultures expressed CD56, thus confirming their myogenic lineage, with no morphological differences between cell groups (**Figure 2B**). However, the T2DM cultures analyzed showed that a subpopulation of cells varied in their CD56 expression, suggesting that CD56 might be differently regulated in the T2DM group (Supplementary Figure 1).

Single cells were then isolated using Fluidigm C1 IFC microfluidic chips. Rates of capture for each chip and visual scoring for each cell are summarized in **Table 2**. Subsequent qPCR analysis showed that each miRNA was expressed in varying fractions of cells ranging from 0 to 100% of cells but were generally found to be comparable between healthy and T2DM groups (**Figure 3A**). Interestingly, 5 miRNAs were detected in notably differing percentages of cells between groups: miR-29b was detected in ∼21% of T2DM cells but only in 5% of healthy cells, whereas miR-106b was detected in 51% of healthy cells versus 40% in the T2DM group (**Figure 3A**). Similarly, miR-143 and miR-145 from the miR-143/145 cluster were detected in 20% of healthy cells versus 11% of T2DM, and 56 % of healthy cells versus 43% of T2DM cells, respectively, whereas miR-138 was detected in 66% of healthy cells versus 51% in the T2DM group.

Applying a principal component analysis (PCA), we observed that miRNA expression profiles could be divided into 4 groups: one cluster of miRNA expression profiles representing most of the healthy cells had a near-complete overlap with a cluster comprising the majority of T2DM cells (T2DM group 1), suggesting that single-cell miRNA expression profiles are largely similar between these groups (**Figure 3B**). Another separate cluster (Mixed) contained cells from both groups, indicating the presence of a subpopulation of cells with a distinct miRNA expression profile not related to T2DM status (**Figure 3B**). Finally, we observed a third cluster exclusively containing T2DM cells (T2DM group 2) (**Figure 3B**), indicating that this subpopulation, defined by a distinct miRNA expression profile, is present in the T2DM group only. This subpopulation did not include cells from subjects that were under medical treatment, described in the method section (Human Subjects).

Applying PCA to the diabetes cells only, we found that formation of the T2DM group 2 subpopulation was largely driven by cells from a single donor, however, other cell donors also contributed to a lesser degree (Supplementary Figure 2). Interestingly, unsupervised clustering analysis revealed that miR-29 family members miR-29a, miR-29b, and miR-29c were all upregulated in the T2DM group 2 cell subpopulation (**Figure 3C** and **Table 3**). Several miRNA transcriptional clusters were also present within the T2DM group 2 subpopulation, including the miR-193b/365 locus, the miR-199/214 locus, as well as miR-24 and miR-27b from the miR-23b/27b/24 locus, and miR-17 and miR-19b from the miR-17 locus. Moreover, although the healthy and T2DM groups largely overlapped, a number of miRNA were also differentially regulated between these groups, namely, miR-24, miR-29a, miR-17, and miR-145 (**Figure 3C** and **Table 3**).

**FIGURE 4:**
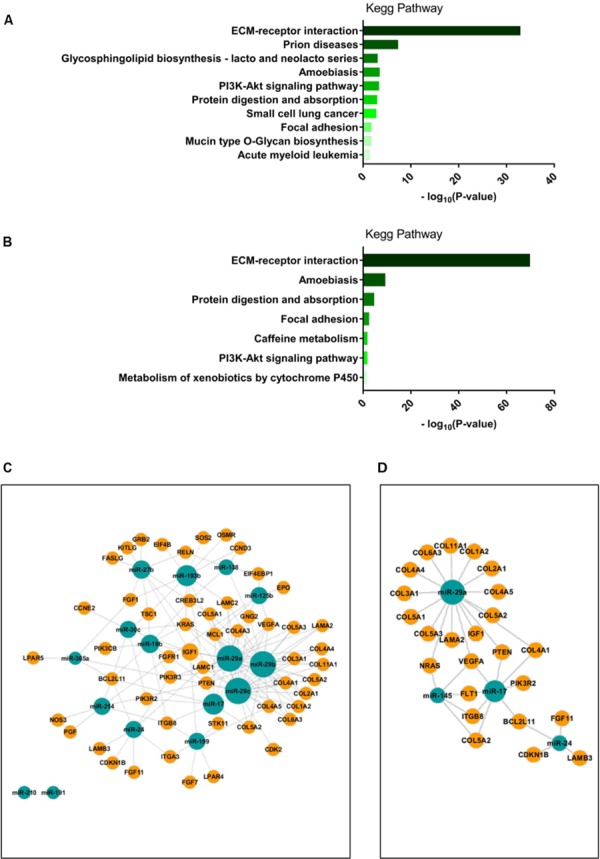
**(A,B)**: miRNA target pathway predictions were performed using Diana miRPath software (Vlachos et al., 2012). Pathway predictions for differentially expressed miRNA between T2DM group 1 and T2DM group 2 clusters are shown in **(A)**; pathway predictions for differentially expressed miRNA between Healthy and T2DM group 1 clusters are shown in **(B)**. Visualization of miRNA-gene interactions were performed using the CytoScape app CyTargetLinker (Shannon et al., 2003; Kutmon et al., 2013). Interactions for differentially expressed miRNA between T2DM group 1 and T2DM group 2 clusters with genes within the PI3K/Akt signaling pathway are shown in **(C)**; interactions for differentially expressed miRNA between Healthy and T2DM clusters with genes within the PI3K/Akt signaling pathway are shown in **(D)**. miRNA node size indicates number of interactions with target genes.

To identify potential target genes of the differentially expressed miRNA, we next applied a pathway prediction analysis using the Diana miRPath tool (Vlachos et al., 2012). The pathway prediction revealed that miRNAs differentially expressed between the two diabetes cell clusters or between the healthy and T2DM clusters were predicted to target several pathways, with the ECM-receptor interaction in the KEGG pathway database being the most likely to be targeted by either group of miRNAs (**Figures 4A,B**). Interestingly, the PI3K/Akt pathway was also a highly likely candidate pathway to be targeted by either group of miRNAs (**Figures 4A,B**) The PI3K/Akt pathway is directly regulated by insulin signaling (Boucher et al., 2014) and has been shown to be dysregulated in skeletal muscle of people with T2DM (Krook et al., 1998, 2000); we therefore chose to examine which genes within this pathway would be targeted by the differentially expressed miRNAs. miRNA-mRNA target analysis of differentially expressed miRNA between the two diabetes clusters showed that 53 genes within the PI3K/Akt pathway were predicted targets of these miRNA and identified miR-29a, miR-29b, and miR-29c as major regulatory nodes (**Figure 4C**). Three of the four differentially expressed miRNA between the healthy and T2DM group 1 clusters overlapped with the differentially expressed miRNA between the two diabetes clusters, namely miR-29a, miR-17, and miR-24, which were predicted to target 24 genes within the PI3K/Akt pathway; similarly, miR-29a showed the highest degree of regulation of these miRNA (**Figure 4D**). Interestingly, among the genes targeted by both subgroups of miRNAs were genes previously described in T2DM, including PTEN, IGF1, and VEGFA (Benjamin, 2001; Schneider et al., 2011; Wu et al., 2011; Pal et al., 2012).

**FIGURE 5:**
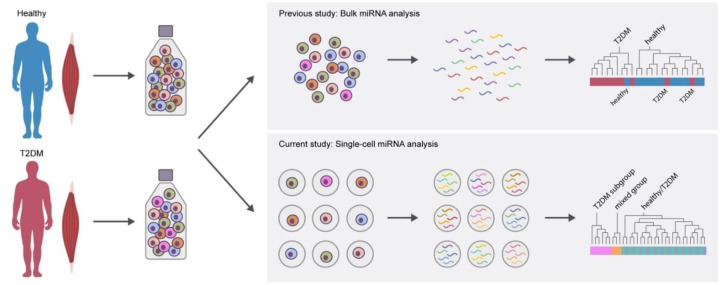
Schematic summary of key findings of the present study. In a previous study using bulk miRNA analysis, a limited number of differentially expressed miRNA were identified in isolated T2DM muscle precursor cells. The present study examined the single-cell expression of miRNA and found that a subgroup of T2DM muscle precursor cells had a distinct miRNA expression profile.

These data thus suggest that although most T2DM cells share miRNA expression profiles with healthy cells, a subpopulation of severely dysregulated muscle precursor cells reside within the muscle progenitor T2DM cells (**Figure 5**).

## Discussion

While skeletal muscle plays a central role in the pathophysiology of T2DM, the role of muscle precursor cells in counteracting or maintaining a disease phenotype is poorly investigated. We hypothesized that a sub-set of muscle precursor cells could be severely dysregulated and were interested in the miRNAs, which are potent regulators of cell differentiation, development, and metabolism. To assess potential miRNA defined cellular subpopulations, we performed miRNA profiling at a single cell level on CD56+ human muscle precursor cells from healthy subjects and individuals with T2DM.

We identified two sub-populations of muscle precursor cells derived from healthy subjects. Interestingly, a distinct third sub-population was detected in cells from people with T2DM. This T2DM-specific subpopulation was characterized by a high expression of miR-29, previously reported as upregulated in T2DM in muscle (Massart et al., 2017). Moreover, miR-29b was detected in 21% of the cells derived from subjects with T2DM compared to only 5% of the cells derived from healthy subjects, further emphasizing the disease-association of mir-29 in the muscle precursor cells.

The advantage of performing single cell analysis becomes clear when comparing our current data with a recent study where we identified miR-23b and miR-27b as downregulated in muscle precursor cells and *in vitro* differentiated myotubes derived from people with T2DM (Henriksen et al., 2017). In our previous study, miR-24, which is transcribed from the same locus as miR-23b and miR-27b, was not identified as differentially expressed in the microarray analysis. Recent advances in single-cell-based analyses have made it increasingly clear that stem cell populations are not homogenous entities (Kumar et al., 2014; Wilson et al., 2015). We therefore hypothesized that subpopulations of T2DM muscle stem cells would have miRNA expression profiles distinct from healthy cells, and that measuring the miRNA expression in “bulk samples” might mask underlying subpopulation trends.

Indeed, in the current study, using a single-cell approach we identified miR-24 as the most distinctly regulated miRNA between muscle precursor cells from healthy donors compared to T2DM donors. However, miR-24 was upregulated in the T2DM specific subpopulation while being downregulated in the T2DM population that mostly overlapped with cells from healthy donors/subjects. Thus, the data presented here demonstrates that two subpopulations of T2DM muscle cells express miR-24 to a highly different degree and measuring an average miR-24 expression of these two populations might therefore yield a result that represents neither subgroup, which could explain why miR-24 was not found to be significantly downregulated in our previous study (Altschuler and Wu, 2010; Henriksen et al., 2017).

Although the applied single-cell approach has its clear advantages in terms of highlighting cellular heterogeneity, there are certain drawbacks of this method to consider when interpreting the data. A specific aim of the present study was to investigate the single-cell expression of a group of previously described miRNA (Henriksen et al., 2017). However, the commercially available pool of primers we used for miRNA pre-amplification did not cover all these miRNAs, and we were thus only able to measure the single-cell expression of some of the previously identified miRNA. Functional implications of our findings in terms of which specific miRNA that are differentially regulated in T2DM cells might therefore be difficult to conclude.

Moreover, the minuscule amounts of cellular material present when working with single cells might be below the detection limit of the primer assays we used (Stahlberg et al., 2013). For example, we previously found miR-23b to be one of the most differentially regulated miRNA between healthy and T2DM groups over time (Henriksen et al., 2017), whereas in this study miR-23b expression was only detected in a small fraction of cells (data not shown). This discrepancy might be a technical issue due to failure of our primer assays to bind to the small amount of miRNA present. A final issue to consider is that the data presented here was not normalized to an endogenous control miRNA. Instead, we made the assumption that the single cell input would serve as a basis for normalization. Normalizing on a per-cell basis has been described as a superior method for normalization in single-cell studies to using an endogenous reference gene (Stahlberg et al., 2013), however, the optimal normalization approach can be debated.

One issue to consider in the present study is that all donors included in the study were not represented in the T2DM group 2 subcluster. Rather, the T2DM group 2 cells came from 3 out of the 5 diabetic donors with most cells being from one individual donor, thus the observed heterogeneity in miRNA expression may also vary between cell donors. Nevertheless, as capture rates also varied considerably between each IFC chip (**Table 2**), we may have failed to isolate cells belonging to the T2DM group 2 cluster due to a low overall capture rate in cells from these donors.

The most striking biological finding in the current study was the identification of a miRNA-defined T2DM-specific subpopulation of muscle precursor cells along with a T2DM-dependent dysregulation of three members of the miR-29 family: miR-29a, miR-29b, and miR-29c. These miRNAs are transcribed from two different genomic loci but possess the same seeding sequence, and are thus predicted to target the same mRNA molecules. These findings are interesting to interpret in light of a previously performed meta-analysis of tissue miRNA expression in models of diabetes, identifying miR-29 as the most upregulated miRNA across different insulin-responsive tissues (Zhu and Leung, 2015). Furthermore, Massart et al. (2017) recently showed that miR-29a and miR-29c were increased in skeletal muscle tissue from patients with T2DM and regulated insulin-mediated glucose metabolism, and miR-29b has similarly been shown to be increased in T2DM muscle (He et al., 2007; Gerlinger-Romero et al., 2017). Thus, there is strong evidence in the literature for an association between T2DM and increased miR-29 expression.

We found that miR-29a, miR-29b, and miR-29c were all upregulated in the T2DM-specific subpopulation, consistent with the previous literature on miR-29 expression being increased in T2DM. Moreover, two other miRNAs were found to be differentially expressed either between the healthy and T2DM group 1 clusters or the two diabetes clusters, namely miR-17 and miR-24. Both of these miRNA have been shown to be regulated by glucose levels (Xiang et al., 2015; Dong et al., 2016) and their concentrations are increased in patients with T2DM (Villard et al., 2015); thus, these two miRNA may represent novel markers of T2DM in skeletal muscle. Interestingly, we demonstrate that these changes are manifested already in muscle precursor cells and are specifically accumulated in a subset of cells, suggesting that muscle precursor cells are affected by and/or contribute to the T2DM muscle phenotype to varying degrees.

Another interesting finding was that although most miRNA were expressed in comparable fractions of cells between groups, miR-29b was only detected in 5% of the muscle precursor cells from healthy individuals, whereas 21% of the muscle precursor cells derived from people with type 2 diabetes were expressing this miRNA, further supporting a role of miR-29 in T2DM disease phenotype, as well as the establishment of T2DM at muscle precursor cell level. Some of the other miRNAs including miR-106b, miR-138, miR-143, and miR-145 were modestly less detected in the cells derived from people with T2DM compared to healthy controls. Although these differences could be technical and therefore should not be over interpreted, these miRNAs have also all been described to be regulated in model systems of muscle insulin resistance or T2DM (Blumensatt et al., 2013, 2014; Zhang et al., 2013; Riches et al., 2014) or glucose uptake and insulin sensitivity (Jordan et al., 2011; Zhou et al., 2016), suggesting a functional relevance of this finding.

Finally, in addition to the T2DM-specific sub-population, we identified another separate cluster of cells comprising a mixture of healthy and T2DM cells. Given that our muscle precursor cells were sorted for CD56 positivity, these cells might represent a subtype of muscle precursor cells common to both groups. However, we cannot exclude the possibility that this cluster is due to presence of a non-muscle cell type, such as fibroblasts. Interestingly, it was recently demonstrated that fibroblasts contribute support during myogenic differentiation (Mackey et al., 2017) bringing this cell population also to interest when comparing isolated cells from healthy subjects and subjects with T2DM.

## Conclusion

We here show that muscle precursor cells derived from humans with T2DM have subpopulations with distinct miRNA expression profiles. These data indicate that that only a subgroup of T2DM muscle precursor cells differ in expression of diabetes-associated miRNA, which may reflect a heterogeneous disease phenotype in T2DM muscle cells. The data presented here thus provides a novel conceptual basis for understanding the muscular phenotype of T2DM.

## Author Contributions

SN designed the study with conceptual advice from CS. SN directed the study. TH, SN, NH, and SH collected and analyzed the data. TH, SN, and CS drafted the manuscript. All the authors interpreted the data, and edited and approved the final version of the article.

## Conflict of Interest Statement

The authors declare that the research was conducted in the absence of any commercial or financial relationships that could be construed as a potential conflict of interest.
