# Supplementary material for: Single Cell Analysis Identifies the miRNA Expression Profile of a Subpopulation of Muscle Precursor Cells Unique to Humans With Type 2 Diabetes
Source: Front Physiol. 2018 Jul 11;9:883. doi: 10.3389/fphys.2018.00883 (PMC6050405; doi:10.3389/fphys.2018.00883)
Supplement: Supplementary file 1 [file Data_Sheet_1.PDF]

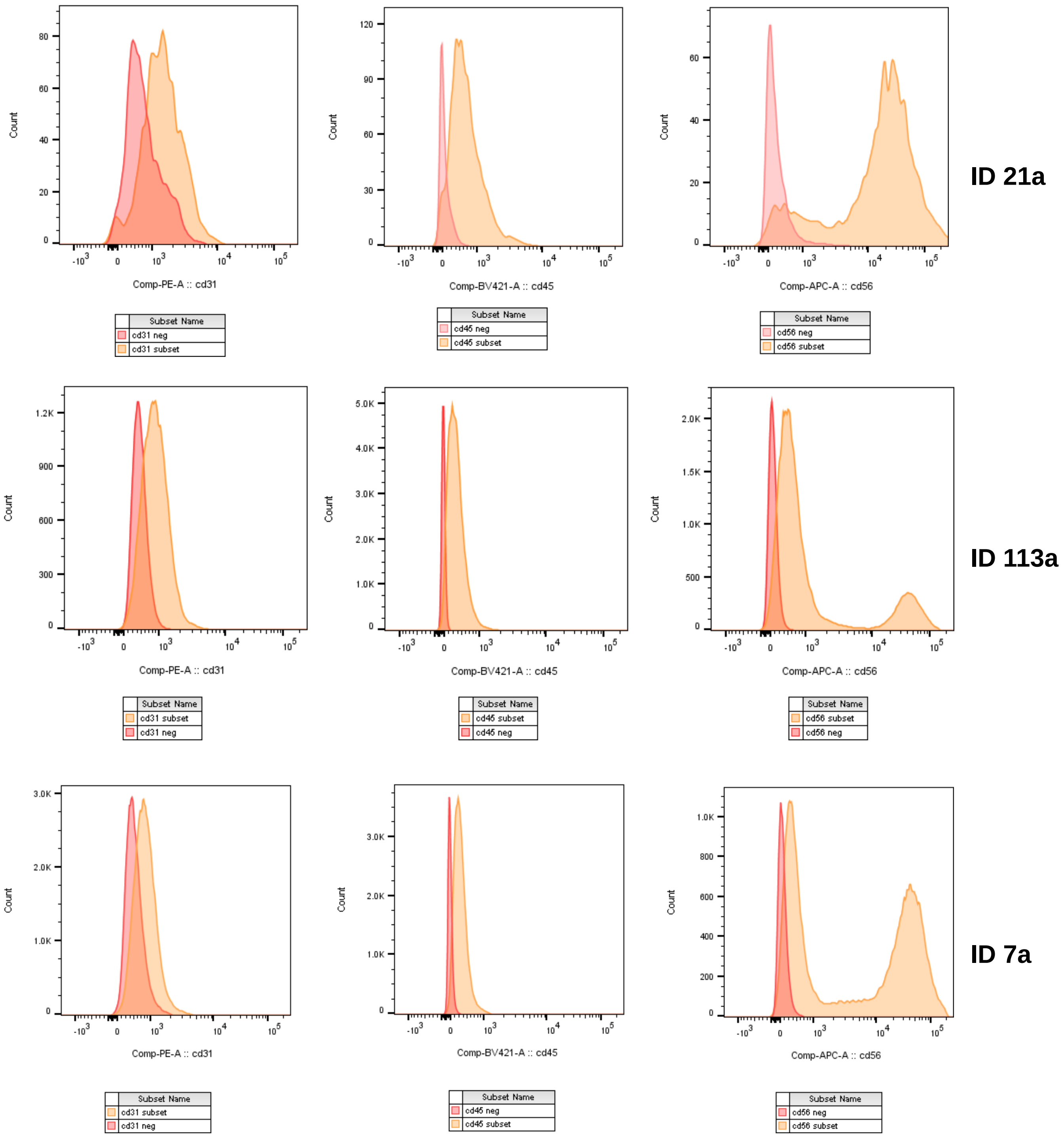

Supplementary figure 1: FACS analysis histograms showing positive (orange) and negative (red) subsets in muscle precursor cells: CD31 conjugated to PE; CD45 conjugated to BV421 and CD56 conjugated to APC.

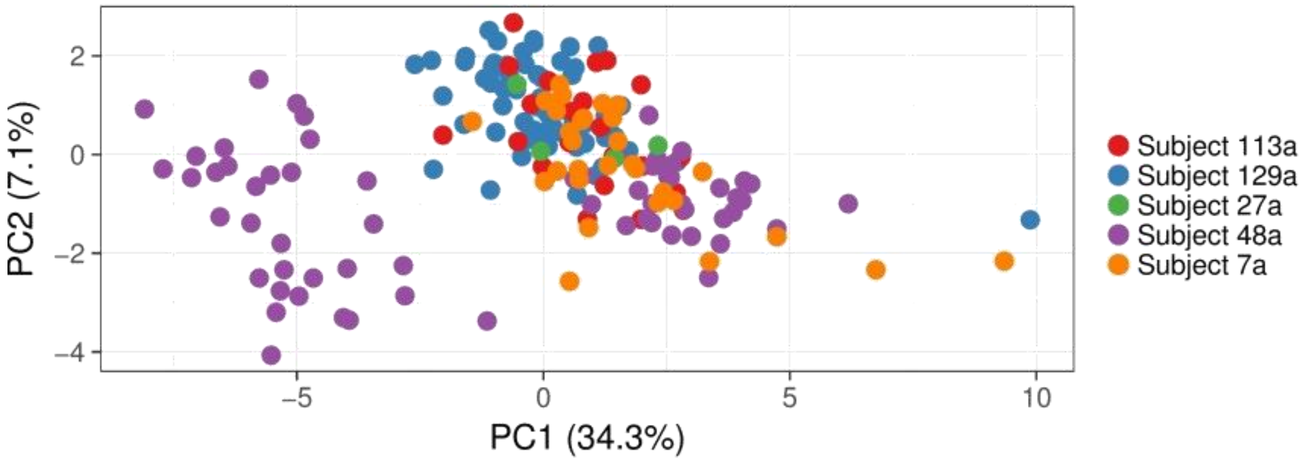

Supplementary figure 2: Principal component analysis of single-cell miRNA expression of all T2DM cells.
